# Top-*k* Shuffled Differential Privacy Federated Learning for Heterogeneous Data

**DOI:** 10.3390/s25051441

**Published:** 2025-02-26

**Authors:** Di Xiao, Xinchun Fan, Lvjun Chen

**Affiliations:** College of Computer Science, Chongqing University, Chongqing 400044, China; fanxc@stu.cqu.edu.cn (X.F.); clj@cqu.edu.cn (L.C.)

**Keywords:** federated learning, data heterogeneity, shuffle model, communication costs

## Abstract

Federated learning (FL) has emerged as a promising framework for training shared models across diverse participants, ensuring data remains securely stored on local devices. Despite its potential, FL still faces some critical challenges, including data heterogeneity, privacy risks, and substantial communication overhead. Current privacy-preserving FL research frequently fails to tackle complexities posed by heterogeneous data adequately, hence increasing communication expenses. To tackle these issues, we propose a top-*k* shuffled differential privacy FL (TopkSDP-FL) framework tailored to heterogeneous data environments. To address the model drift issue effectively, we design a novel regularization for local training, drawing inspiration from contrastive learning. To enhance efficiency, we propose a bidirectional top-*k* communication mechanism that reduces uplink and downlink overhead while strengthening privacy protection through double amplification with the shuffle model. Additionally, we shuffle all local gradient parameters at the layer level to address privacy budget concerns associated with high-dimensional aggregation and repeated iterations. Finally, a formal privacy analysis confirms the privacy amplification effect of TopkSDP-FL. The experimental results further demonstrate its superiority over other state-of-the-art FL methods, with an average accuracy improvement of 3% compared to FedAvg and other leading algorithms under the non-IID scenario, while also reducing communication costs by over 90%.

## 1. Introduction

With the arrival of 6G, its widespread deployment will significantly increase network connection density, further pushing the scale of Internet of Things (IoT) devices. These smart devices (e.g., wearables, smart home systems, and industrial sensors) will continue to generate massive amounts of data, making methods of efficiently processing and utilizing these data an urgent challenge. At the same time, the exponential growth of IoT devices will exacerbate resource allocation and energy consumption issues [1], placing higher demands on the existing computing and communication infrastructure. However, traditional machine learning approaches have inherent limitations in data privacy and security, making it difficult to adapt to such a highly distributed data environment. To address this challenge, federated learning (FL) [2] has emerged as a decentralized computing paradigm that enables cross-device collaborative training without exposing individual client data, thus facilitating data sharing and model optimization while safeguarding privacy. Therefore, FL shows a broad application prospect in the intelligent computing system in the 6G era. However, in practical applications, especially in IoT scenarios, FL still faces many challenges, mainly focusing on three key aspects: data heterogeneity, communication efficiency, and privacy preservation.

In practical applications, data heterogeneity, also known as non-independent and identically distributed (non-IID) data, is a key challenge of FL. Such heterogeneity causes discrepancies between the optimization goals of local models and the overall global objective, thereby affecting the performance and stability of the global model while making the convergence process more complex. To tackle this issue, existing studies are mainly divided into two topics: global aggregation optimization and local training modification.

For global aggregation optimization, researchers have focused on improving server-side update strategies to ensure that global model updates are more effectively aligned with the overall optimization objective. For example, FedAvg [2] implements a weighted average of model parameters according to client data sizes, establishing the fundamental framework of FL. However, under non-IID conditions, when the number of local training iterations grows, the difference between local and global model objectives will be intensified, thereby hampering convergence efficiency. To address this, FedAdp [3] dynamically adjusts client weights by analyzing the angle between local and global gradients, reducing communication rounds. Similarly, other methods [4,5] refine the aggregation processes to enhance alignment with the global optimum. However, these approaches typically require access to a unified dataset to accurately ascertain the positioning of local models within the probability space. This dependency imposes substantial limitations, particularly in resource-constrained environments where access to such datasets is restricted or infeasible. In contrast, local training modifications [6,7,8] aim at introducing regularization strategies into the local optimization process, effectively reducing the divergence between local updates and the global objective. These approaches exhibit high adaptability and efficiency, requiring only minimal modifications to the original FL algorithm, thereby mitigating the overall system burden. For example, SCAFFOLD [7] utilizes control variables to correct update directions and improve convergence accuracy, albeit at the expense of increased communication overhead. Similarly, MOON [8] applies a contrastive learning framework to align local models with the global model but struggles to handle highly heterogeneous data. Consequently, there remains a lack of scalable solutions that can simultaneously address data heterogeneity, maintain efficiency, and deliver strong performance.

In addition to data heterogeneity, communication efficiency is also crucial in FL, mainly because of the frequent exchange of model updates between clients and the server, which significantly consumes network bandwidth and device energy. This challenge is especially critical in IoT scenarios with limited resources. To mitigate this burden, two common optimization techniques are often employed: quantization and sparsification. Quantization reduces communication overhead by converting model parameters from high precision (e.g., 32-bit floating point) to lower precision (e.g., 8-bit floating point). For instance, SignSGD [9] binarizes gradients, transmitting only their signs to achieve high compression rates, while TernGrad [10] employs stochastic quantization, restricting gradient values to three discrete levels (0, 1, −1). However, these approaches often face challenges with performance degradation, particularly in non-IID settings, due to the inherent loss of precision. Sparsification focuses on transmitting only the critical gradient components to reduce communication overhead. For example, Heafield et al. proposed the top-*k* selection method, which selects the gradients with the most significant absolute values to minimize data transmission without significantly impacting model performance [11]. Similarly, N. Strom et al. [12] proposed transmitting gradients that exceed a predetermined threshold while aggregating residuals for smaller gradients to ensure update accuracy. Further advancements, such as deep gradient compression [13], combine sparsification with techniques like gradient clipping and momentum correction to improve compression efficiency. To enhance adaptability, Han et al. [14] introduced a dynamic sparsification method that adjusts the sparsity level in real time, significantly improving both communication and computational efficiency. Although quantization and sparsification techniques have achieved significant progress in reducing communication costs, both approaches face limitations in highly heterogeneous and large-scale FL settings. Therefore, enhancing the efficiency of both uplink and downlink communication while maintaining model accuracy and convergence remains a critical area of research for FL, particularly in IoT scenarios.

Privacy leakage remains a significant challenge in FL. Although FL effectively avoids direct data leakage by keeping data locally, the model updates sent by participants may still be vulnerable to inference attacks. This issue is particularly prominent in IoT environments, where the high-dimensional data generated by devices and the multiple iterations required in training accelerate the consumption of privacy budgets, further threatening the privacy of the training process. Current privacy-preserving techniques primarily include homomorphic encryption (HE), secure multi-party computation (SMC), and differential privacy (DP). HE protects data privacy by enabling direct algebraic operations on encrypted data. However, its significant computation and communication overhead render it unsuitable for resource-limited IoT environments [15]. SMC ensures secure computation by enabling multiple parties to collaborate without revealing their private data. While it offers a high level of security, its complex implementation poses significant challenges, limiting its scalability in large-scale distributed systems [16,17]. In contrast, DP is widely adopted due to its lower computational cost and scalability. Centralized DP (CDP) protects privacy by adding noise to aggregated gradients or model parameters, but it relies on trusted servers and may not fully safeguard user data in decentralized environments [18]. Local DP (LDP), on the other hand, eliminates the need for a trusted server by adding noise before data is uploaded by clients, though it often sacrifices model accuracy [19]. To address the balance between privacy preservation and performance, Google designed the Encoder-Shuffler-Analyzer (ESA) framework [20] with a shuffler to achieve the privacy preservation effect of LDP and the accuracy of CDP. Erlingsson et al. [21] demonstrated that the shuffle model enhances the level of preservation through the privacy amplification effect. Girgis et al. [22] analyzed the privacy amplification effect of stochastic gradient descent and client self-sampling DP. However, these studies overlook the issue of privacy budget explosion that arises after multiple iterations.

Although existing research has made some progress in communication optimization and privacy preservation, it often neglects the influence of non-IID data on model performance and convergence efficiency. Data heterogeneity not only exacerbates model bias, but also significantly degrades global model performance and increases communication costs. Therefore, it is particularly important to design a framework that can effectively mitigate data heterogeneity and reduce communication overhead while preserving privacy. We propose a TopkSDP-FL framework for data heterogeneity scenarios that achieves a balance among model performance, communication cost, and privacy.

Specifically, the key contributions of this paper are summarized as follows:

- We design an innovative regularization term inspired by contrastive learning, which effectively exploits the relationship between local and global models to adjust local update directions, thereby improving convergence speed and enhancing overall model performance.
- We propose a bidirectional top-*k* communication mechanism integrated with the shuffle model, achieving dual privacy amplification while significantly reducing communication overhead in both uplink and downlink directions.
- We develop a layer-wise parameter shuffling strategy to mitigate the issue of privacy budget exhaustion. This approach enhances the anonymity of local models and ensures robust privacy protection during iterative updates.
- Comprehensive experiments demonstrate that the TopkSDP-FL framework achieves superior performance accuracy and communication efficiency. Furthermore, theoretical analysis validates its effectiveness in achieving privacy amplification.

The structure of this paper is as follows: Section 2 provides the preliminary of FL and DP. Section 3 details the proposed TopkSDP-FL framework. Section 4 presents a comprehensive privacy analysis of TopkSDP-FL. Section 5 discusses the simulation results. Finally, Section 6 concludes this paper.

## 2. Preliminary

### 2.1. Federated Learning

**Figure 1:**
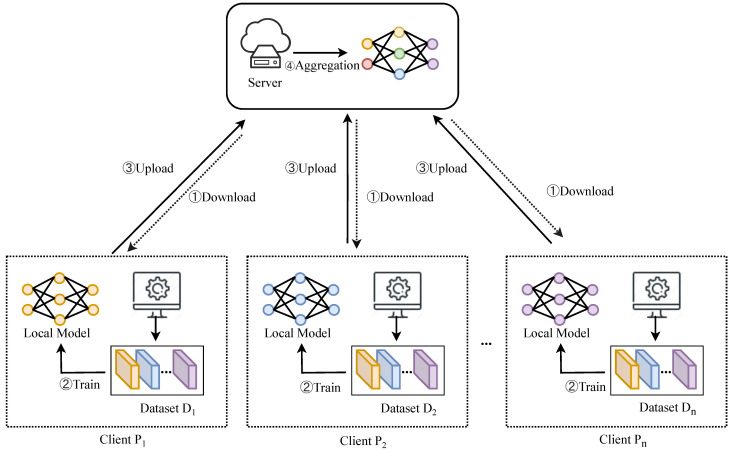
The basic framework of federated learning.

The purpose of FL is to train a global model using a server and multiple clients. As illustrated in Figure 1, assume there are N clients, denoted by $\left\{P_{1},P_{2},\ldots ,P_{N}\right\}$, and $D_{i}$ denotes the dataset owned by each client; $P_{i}$, $w_{i}^{t}$ denotes the local model of client $P_{i}$ in the *t*-th round, and $w_{g}^{t}$ denotes the global model in the *t*-th round.

First, the server distributes the global model to each client. Second, clients use the latest model to update the local model by training on their local dataset:

(1) $$w_{i}^{t}=w_{i}^{t-1}-\alpha \nabla L\left(f\left(x^{i},w_{i}^{t-1}\right),y^{i}\right).$$

After local training, the latest local model is uploaded to the server. Then, the server performs the following aggregation operation:

(2) $$w_{g}^{t+1}=\sum_{i=1}^{N}\frac{|D_{i}|}{\sum_{k}\left|D\right|}w_{i}^{t}.$$

Finally, the latest global model is then distributed, and the above process is repeated until the final model converges. The optimization goal of FL is to obtain an optimal global model by collaborative training on the datasets $D=\{D_{1},D_{2},\ldots ,D_{k}\}$:

(3) $$O\left(w_{g},D\right)=\min_{w^{i}}\;E_{D_{j}∼\tilde{D}}L\left(w^{i},D_{i}\right),i\in \left[1,N\right],$$

where $L\left(w^{i},D_{i}\right)$ denotes the empirical loss function, and $\tilde{D}$ denotes the distribution of *D*. In reality, however, the data distribution $\tilde{D}$ may be unbalanced across clients. Local models tend to overfit these unbalanced data, thus affecting the global model performance.

### 2.2. Differential Privacy

DP can provide a rigorous mathematical foundation for data privacy, defined by Dwork [23] as follows:

**Definition 1**  (DP)**.** *For a randomized algorithm M, if for any two neighboring datasets $D,D^{\prime }\in X^{n}$ that differ on only one sample, the probability of getting any same output set O is satisfied, as follows,*

(4) $$Pr\left[M\left(D\right)\in O\right]\leq e^{\epsilon }Pr\left[M\left(D^{\prime }\right)\in O\right]+\delta ,$$

*then the algorithm M satisfies (ϵ,δ)-DP.*

These CDP approaches rely on the assumption of a trusted server to perform model aggregation. However, this assumption is often difficult to realize in practice. This prompts the use of LDP, where the user achieves privacy protection using random perturbation before the data is transmitted. In Definition 1, when δ=0, the framework satisfies pure DP. Otherwise, it is referred to as approximate DP, where ϵ represents the privacy budget and δ quantifies the relaxation level of DP.

However, the LDP mechanism adds more noise compared to CDP, resulting in a degradation of model performance. In order to implement LDP with less perturbation, the SDP was born [20]. The SDP consists of three parts: the encoder (R), the shuffler (S), and the analyzer (A), which ultimately satisfy DP.

**Definition 2**  (SDP [21])**.** *Given n users, each user has one data point $x_{i}$. Let $R:X\to Y^{m}$ denote the random perturbation of each user’s data point to achieve $\epsilon_{l}$-LDP, $S:Y^{*}\to Y^{*}$ denotes the execution of the shuffle operation, and $A:Y^{*}\to Z$ analyzes and computes to obtain the final result. Therefore, the SDP protocol can be expressed as P=(R,S,A). When R satisfies $\epsilon_{l}$-LDP, according to the shuffling privacy amplification theorem, $S\circ R=S(R\left(x_{1}\right),\ldots ,R\left(x_{n}\right))$ satisfies $\left(\epsilon_{c},\delta_{c}\right)$-DP. The protocol P thus also satisfies $\left(\epsilon_{c},\delta_{c}\right)$-DP of the post-processing property of DP, where $\epsilon_{c}<\epsilon_{l}$. Consequently, the SDP requires less perturbation than the LDP.*

## 3. Proposed Method

**Figure 2:**
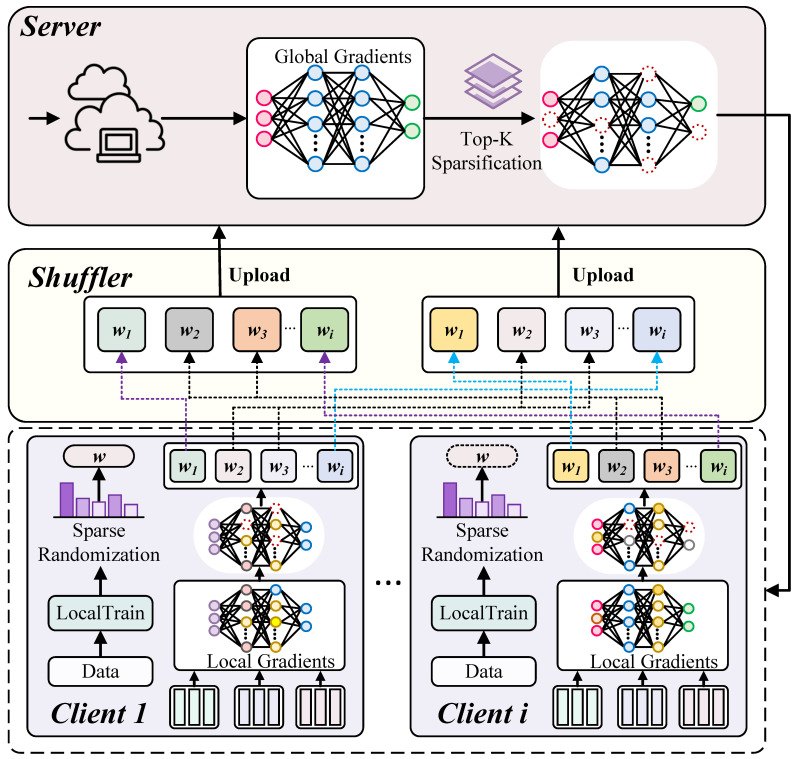
The workflow of TopkSDP-FL.

To tackle the challenges in FL, we propose the TopkSDP-FL method, focusing on improving model accuracy, optimizing communication efficiency, and strengthening privacy protection, specifically for non-IID scenarios in resource-constrained environments. Figure 2 illustrates the overall framework of TopkSDP-FL, which consists of the following steps: (1) global model initialization, (2) local training, (3) gradient sparsification and randomization, (4) parameter shuffling, and (5) gradient aggregation and sparsification. The following subsections provide a detailed description of each step.

### 3.1. Global Model Initialization

Before the training starts, the server initializes a global model $w_{0}^{t}$ and a set of hyperparameters required during the training process, such as the total communication rounds *T* and the learning rate η. The server also collaborates with the clients to determine an initial weight range based on prior knowledge, which helps maximize the model’s convergence during training. Using this range, the server finalizes the initialization of the global model $w_{0}$. Afterward, the server distributes the initial global model $w_{0}$ along with some hyperparameters to clients participating in local training.

### 3.2. Local Training and Gradient Computation

In the *t*-th training round, each client receives the latest global model $w_{g}^{t}$ from the server and initializes its local model as $w_{i}^{t}=w_{g}^{t}$, replacing the previous local model $w_{i}^{t-1}$. The client then performs local training based on its local dataset $D_{i}$, executing *E* steps of local optimization. During each local epoch, the client splits its dataset into multiple mini-batches by size *b*, and for each mini-batch, the model is updated using gradient descent as follows:

(5) $$w_{i}^{t,e+1}=w_{i}^{t,e}-\eta \nabla \ell \left(w_{i}^{t,e};B\right),$$

where η is the learning rate, *ℓ* is the local loss function, *e* represents the current local epoch, and *B* denotes a mini-batch.

In FL, due to the typically non-IID nature of client data, local model updates often deviate from the global optimization objective, causing model drift. This drift not only weakens the global model’s generalization ability, but may also introduce systematic bias, particularly in high data heterogeneity, significantly impacting the overall model performance of FL.

**Figure 3:**
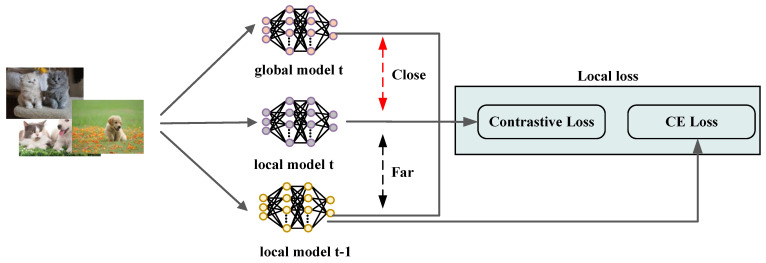
The framework of the contrastive loss function in TopkSDP-FL.

Currently, many approaches aimed at addressing the model drift problem exhibit inherent limitations. Many existing solutions focus only on the relationship between local model updates and global model updates; however, the dynamic association between successive rounds of local model updates is ignored. This neglect of the relationship between successive local model updates may lead to an inability to adequately capture the evolutionary characteristics of the local model during model iterations, which in turn affects the overall performance of the model. In addition, the effectiveness of these methods is significantly reduced in highly heterogeneous data scenarios, making it difficult to cope with the challenges posed by high data heterogeneity. To solve this problem, we propose a new local loss function inspired by contrastive learning, which is illustrated in Figure 3. In the classical framework of contrastive learning, two different augmented views are usually generated for each image as positive sample pairs, while at the same time, other images are randomly selected from the dataset to construct negative sample pairs. The core of the mechanism is to guide the model so that the positive sample pairs are close to each other in the feature space, while the negative sample pairs are far away from each other.

Inspired by this idea, we applied the contrastive approach to the model training process. Specifically, we treated the current global model $w_{g}^{t}$ as a positive sample and the local model $w_{i}^{t-1}$ from the previous round as a negative sample. The goal was to enhance the consistency between the current local model $w_{i}^{t}$ and the global model $w_{g}^{t}$, optimizing the process such that the distance between the two in the parameter space was minimized. Simultaneously, we aimed to increase the distance between the current local model $w_{i}^{t}$ and the previous round’s local model $w_{i}^{t-1}$, thereby reducing the reliance on the historical model state. This design ensures that the update direction of the local model is closely aligned with the global optimization objective, effectively preventing the local model from deviating from the global optimal solution during the iteration process. As a result, it significantly mitigates the model drift problem. To achieve this, we introduce a specific regularization term into the local loss function, expressed as follows:

(6) $$L_{\mathrm{reg}}=\alpha ∥w_{i}^{t}-w_{g}^{t}{∥}^{2}-\beta {∥w_{i}^{t}-w_{i}^{t-1}∥}^{2},$$

where α and β are hyperparameters controlling the relative importance of the positive and negative samples. This design ensures global consistency in local updates and effectively mitigates model drift.

However, solely relying on the regularization term is insufficient for optimizing classification tasks in supervised learning. To enhance the local model’s performance on its dataset, we also incorporated a cross-entropy loss into the loss function:

(7) $$L_{\mathrm{CE}}=\ell_{\sup}\left(w_{i};\left(x,y\right)\right),$$

where (x,y) represents a local data sample, and $\ell_{\sup}$ is the supervised cross-entropy loss. The inclusion of cross-entropy loss ensures the model effectively captures class information from the local dataset, further improving classification performance. Finally, the complete local loss function is defined as follows:

(8) $$L=L_{\mathrm{CE}}+L_{\mathrm{reg}}.$$

After completing local training, the client computes the model update (or gradient) based on the final local model $w_{i}^{t}$ and the global model $w_{g}^{t}$. The model update is defined as follows:

(9) $$\Delta w_{i}^{t}=w_{i}^{t}-w_{g}^{t}.$$

This optimization process allows the client to fully utilize its local data to improve the local model’s performance while leveraging the contrastive mechanism to enhance the stability and generalization of the global model.

### 3.3. Gradient Sparsification and Randomization

To enhance communication efficiency and strengthen privacy protection, we propose a gradient sparseRand method, detailed in Algorithm 1, which combines the top-*k* sparsification algorithm with a randomization mechanism. First, the gradient updates $\Delta w_{i}^{t}$ are clipped within the range [−C,C] to prevent gradient explosion, and the clipped gradient values are denoted as $c_{i}^{t}$ (Line 1). Next, the top-*k* strategy selects the top-*k* gradient dimensions with the largest absolute values, whose indices are stored in the set $S_{\mathrm{top}}$, while the remaining d−k dimensions denoted as $S_{\mathrm{non}}$ are classified as non-important and set to zero (Lines 2–3). Simultaneously, any gradient components that remain untransmitted are preserved as residuals and accumulated into the subsequent round’s gradient updates, thereby ensuring the integrity of the information. After sparsification, the gradients in $S_{\mathrm{top}}$ are randomized using the Gaussian mechanism $R_{\epsilon_{lk}}$ to satisfy DP, as defined in Equation (10) (Line 5).

(10) $$N(0,\left(\Delta f/\epsilon_{lk}\right)\sqrt{2\log(1.25/\delta )}),$$

where Δf=2C/(n·η) represents the sensitivity, *C* is the clipping threshold, *n* is the number of local data points, η is the learning rate, and $\epsilon_{lk}$ is the privacy budget allocated to the *k*-th dimension. By allocating a higher privacy budget to these important gradients, the noise impact on model performance is significantly reduced. For the gradients in $S_{\mathrm{non}}$, they are directly set to zero, ensuring that no meaningful information is leaked. The sparsified and randomized model updates, $s_{i}^{t}$ along with the residuals $res_{i}^{t}$, are returned. Additionally, subsampling techniques further amplify privacy by enabling less noise to be injected under the same privacy budget, effectively reducing communication costs while improving model performance and strengthening privacy guarantees.    
**Algorithm 1:** SparseRandom algorithm
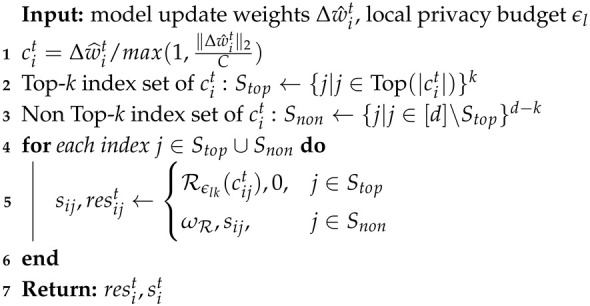


### 3.4. Parameter Shuffling

In FL, the accumulation of privacy budgets over multiple iterations increases the risk of privacy leakage. Furthermore, directly transmitting client updates allows the server to trace updates back to specific clients, compromising anonymity. To address these issues, we propose a layer-based parameter shuffling method, which breaks the direct association between clients and their model updates while reducing the dimensionality of transmitted data. This approach effectively mitigates the accumulation of privacy budgets.

First, each client divides its local model update $s_{i}^{t}$ into smaller submodels according to the layer structure of the model. If the model contains *L* layers, $s_{i}^{t}$ can be split into *L* submodels $\left(s_{i1}^{t},s_{i2}^{t},\ldots ,s_{iL}^{t}\right)$, where each submodel $s_{id}^{t}$ corresponds with the weights of the *d*-th layer. To ensure correct aggregation on the server side, each submodel is indexed to indicate its position in the original model structure. This model splitting process can be represented as follows:

(11) $$\left\{\left(1,s_{i1}^{t}\right),\ldots ,\left(L,s_{iL}^{t}\right)\right\}=\mathrm{split}\left(s_{i}^{t}\right).$$

Next, the client sends these indexed submodel tuples to the shuffler for parameter shuffling. The shuffler randomly rearranges the submodel tuples uploaded by all clients, as follows:

(12) $$\left\{s_{i}^{t}\right\}\leftarrow \mathrm{Shuffling}\left(\left\{\left(1,s_{11}^{t}\right),\ldots ,\left(N,s_{NL}^{t}\right)\right\}\right),$$

where Shuffling(·) is a random permutation function that reorders the submodels. This process ensures that the hierarchical relationship of the layers is preserved while breaking the direct link between each submodel and its original client. For instance, consider three client models $w_{1},w_{2},w_{3}$, each consisting of three layers. After partitioning, their submodel tuples can be represented as follows: $\left\{\left(1,w_{11}\right),\left(2,w_{12}\right),\left(3,w_{13}\right)\right\},\left\{\left(1,w_{21}\right),\left(2,w_{22}\right),\left(3,w_{23}\right)\right\},\{\left(1,w_{31}\right),$

$\left(2,w_{32}\right),\left(3,w_{33}\right)\}.$ After shuffling, these submodels may be reorganized as follows: ${w_{1}}^{\prime }=\{\left(1,w_{21}\right),\left(2,w_{32}\right)$,$\left(3,w_{13})\right\}$, ${w_{2}}^{\prime }=\left\{\left(1,w_{11}\right),\left(2,w_{22}\right),\left(3,w_{33}\right)\right\}$, ${w_{3}}^{\prime }=\{\left(1,w_{31}\right),\left(2,w_{12}\right)$, $\left(3,w_{23})\right\}$.

Finally, the shuffler sends the shuffled models to the server for global aggregation. By decoupling client updates from their identities through shuffling, the proposed method prevents the accumulation of privacy budgets across multiple communication rounds while maintaining the integrity of the global model structure.

### 3.5. Gradient Aggregation and Sparsification

At this stage, the server performs gradient aggregation according to Equation (13), generating the global model update $\Delta w_{g}^{t+1}$. The aggregation operation is defined as follows:

(13) $$\Delta w_{g}^{t+1}=\sum_{i=1}^{N}\frac{|D_{i}|}{\left|D\right|}s_{i}^{t},$$

where $|D_{i}|$ denotes the size of the local dataset of client *i*, |D| represents the total size of all client datasets, and $s_{i}^{t}$ is the shuffled gradient update uploaded by the shuffler in round *t*.

Although the clients sparsify the uploaded information by uploading only the more important parameters, the data distribution of each client is non-IID, so the non-zero elements retained in the model update are different for each client. As the client numbers increase, the model’s non-zero parameters gradually increase after server aggregation, making the global model update denser. To further reduce the communication cost, we utilize the same sparsification method to reduce the downlink communication after aggregation and also introduce the corresponding error correction. This reduces the downlink communication overhead and a portion of the noise interference. Specifically, we transmit only the most important parameters before distributing global model updates, greatly minimizing the volume of transmitted data. The detailed process is outlined as follows:

(14) $$\Delta {\hat{w}}_{g}^{t+1}=\Delta w_{g}^{t}+res_{g}^{t-1},$$

(15) $$s_{g}^{t+1},res_{g}^{t+1}=sparse\left(\Delta w^{t+1},sr\right),$$

where $s_{g}^{t+1}$ is the sparsified global model update, ${res}_{g}^{t+1}$ is the newly stored residuals, and sr is the sparsity ratio, indicating the proportion of zeroed elements in the gradients. Finally, the server distributes the sparsified global model updates $s_{g}^{t+1}$ to the clients. This bidirectional sparsification mechanism on both uplink and downlink communication effectively reduces transmission overhead while maintaining model performance.

### 3.6. TopkSDP-FL Framework

In the TopkSDP-FL algorithm, the server first initializes the global model parameters, as shown in Algorithm 2. During each communication round, each client receives the global model update $s_{g}^{t}$ and initializes the local model $w_{i}^{t}$ (Line 4). The client then performs *E* rounds of local training on its local dataset (Lines 6–13) and computes the gradient update $\Delta w_{i}^{t}$ (Line 14). Next, the client applies the sparseRand operation (Lines 15–16) to protect privacy, generating the sparsified gradient ${\hat{s}}_{i}^{t}$ and residual $res_{i}^{t}$. The sparsified gradient is then split into submodels based on the model’s layer structure and sent to the shuffler (Line 17). The shuffler randomly reorganizes the submodels uploaded by all clients (Line 20), shuffling their sequence to obscure the source of each update before sending the shuffled gradients to the server. The server performs a weighted aggregation of the received gradients to generate the global update $\Delta w_{g}^{t+1}$ (Line 22). To further reduce communication overhead, the server applies sparsification to the aggregated global update, retaining the most important parameters and adding the residual (Lines 23–24). Finally, the server distributes the sparsified global model update to the clients for the next communication round, and the process repeats until the global model converges.
**Algorithm 2:** TopkSDP-FL algorithm
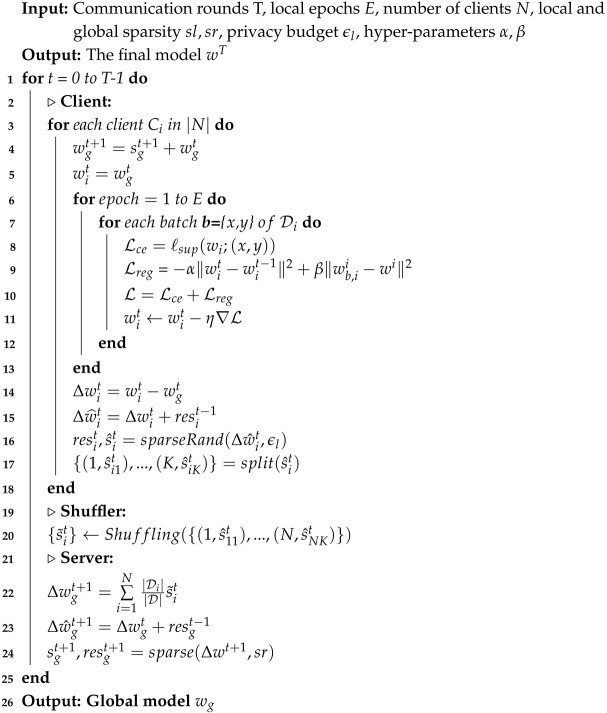


## 4. Privacy Analysis

This section presents a comprehensive privacy analysis of TopkSDP-FL, beginning with an overview of the fundamental privacy protections. We further demonstrate the privacy amplification achieved by our TopkSDP-FL, providing a formal demonstration to validate its efficacy.

In our framework, both clients, the shuffler, and the server, are assumed to follow an honest but curious model. This implies they adhere to the protocol but may still attempt to infer additional privacy-related information from other clients. To mitigate privacy leakage during gradient transmission, each client first applies noise to the gradients before uploading them to satisfy $\epsilon_{l}$-LDP, effectively obfuscating critical data characteristics. The shuffler further enhances privacy by randomly shuffling the received gradients across layers, increasing anonymity and severing the direct link between the gradients and specific clients. The server aggregates the shuffled gradients, with additional privacy measures such as gradient perturbation and parameter shuffling. These privacy-enhancing mechanisms make it difficult for any parties, whether it be the server, shuffler, or external adversaries, to infer sensitive information.

**Theorem 1**  (Shuffle Model [21])**.** *If mechanism R satisfies $\epsilon_{l}$-LDP, where $\epsilon_{l}\leq \log(n/\log\left(\left(1/\delta_{c}\right)\right)/2$, then mechanism M=S∘R satisfies $\left(\varepsilon_{c},\sigma_{c}\right)$-DP:*

(16) $$\epsilon_{c}=O\left(\left(1\wedge \epsilon_{l}\right)e^{\epsilon_{l}}\sqrt{\log(1/\delta_{c})/n}\right).$$

**Theorem 2**  (Subsampling [24])**.** *If mechanism $M:X^{n}\to Y$ satisfies (ε,σ)-DP, then for a subset m≤n, mechanism $M^{\prime }:X^{m}\to Y$ satisfies $\left(\log\left(1+\left(m/n\right)\left(e^{\epsilon }-1\right)\right),\left(m/n\right)\delta \right)$-DP.*

**Proof.**  Assuming the total privacy budget is $\epsilon_{l}$, since only the top-*k* values are perturbed, each dimension can obtain a larger privacy budget $\epsilon_{lk}=\epsilon_{l}/k$. In this scheme, we first use $R_{\varepsilon_{lk}}\left(\cdot \right)$ to randomize each selected data point, then we use S(⋅) to generate a set randomly, and finally we perform the aggregation operation on the server, where the shuffle model and subsampling will affect the privacy budget. Next, we compute the final server implementation of $\left(\epsilon_{c},\delta_{c}\right)$-DP. □

Firstly, we bring $\epsilon_{lk}$ into Theorem 1 to derive the central privacy $\epsilon_{ck}$ after amplification by shuffling, and then according to the subsampling privacy amplification theorem, we bring $\epsilon_{ck}$ into Theorem 2 to get $\left(\epsilon_{cd},\delta_{cd}\right)$:

(17) $$\epsilon_{ck}=O\left(\left(1\wedge \epsilon_{lk}\right)e^{\epsilon_{lk}}\sqrt{\log(\beta /\delta_{cd})/n}\right),$$

(18) $$\epsilon_{cd}=\log\left(1+\beta \left(e^{\epsilon_{ck}}-1\right)\right),\beta =\frac{k}{d}.$$

## 5. Experiments and Result

In this section, we comprehensively evaluate the proposed algorithm in terms of several dimensions, including accuracy, efficiency, and privacy. Specifically, we first introduce the experiment’s basic setup, then show our method’s model performance on different datasets under non-IID settings and compare it with other FL algorithms. We also analyze the communication overhead and computational overhead of each method. Finally, we explore the impact of factors such as sparsity rate, degree of heterogeneity, privacy budget, and hyperparameters on model performance.

### 5.1. Experiment Settings

**Datasets and Baselines.** To investigate the effectiveness of TopkSDP-FL, it is compared with several advanced approaches, including FedAvg [2], FedProx [6], MOON [8], SCAFFOLD [7], and FedADMM [25]. Besides that, SOLO is also introduced as a comparison, in which each client trains only on its own dataset. We choose FedAvg as the baseline, and the other three methods also introduce regular terms or control variables to deal with data heterogeneity. The hyperparameters μ that exist in MOON and FedProx are set to 1 and 0.001, respectively. We validate on three datasets: MNIST, Fashion-MNIST, and CIFAR-10.

**Implementation Details.** For MNIST and Fashion-MNIST, we use a three-layer fully connected neural network. For CIFAR-10, a CNN is used with two convolutional layers and two fully connected layers. We use the SGD optimizer with a momentum of 0.5, a learning rate of 0.01, a batch size of 64, and 200 communication rounds for stable global model convergence. The loss function uses hyperparameters α=0.001 and β=0.005 for MNIST and Fashion-MNIST, and α=0.001 and β=0.001 for CIFAR-10. Both sl and sr are set to 0.9.

**Environment Settings.** We implemented TopkSDP-FL and other methods using PyTorch 2.1.2 with Python 3.10, and executed them on a Linux server equipped with an NVIDIA GeForce RTX 4090 GPU running Ubuntu.

**Figure 4:**
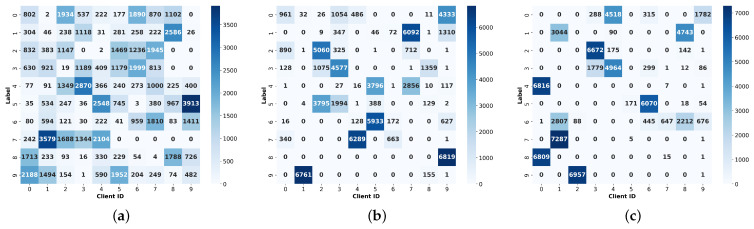
Data distribution heterogeneity with different γ settings. (**a**) γ=0.5; (**b**) γ=0.1; (**c**) γ=0.01.

**Data preprocessing.** This paper focuses on the non-IID data scenario and introduces a label imbalance setting based on the Dirichlet distribution. The proportion of labeled samples is independently assigned to each client by the Dirichlet distribution, and the parameter γ controls the level of data heterogeneity, where the smaller γ, the higher the heterogeneity. We used three settings (γ=0.5, 0.1, 0.01) to simulate data distributions with different degrees of heterogeneity, as shown in Figure 4, to study and compare the performance of different FL algorithms under the non-IID settings.

### 5.2. Accuracy and Efficiency

In this subsection, we first evaluate the accuracy of TopkSDP-FL compared to other methods on various datasets. We then evaluate the communication overhead and computational overhead of our algorithm compared to others, demonstrating its effectiveness in both accuracy and efficiency.

**Figure 5:**
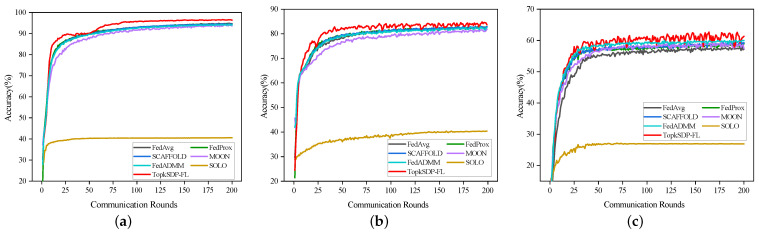
Test accuracy of different schemes on three datasets using six methods (i.e., FedAvg [2], FedProx [6], SCAFFOLD [7], MOON [8], FedADMM [25], SOLO, and TopkSDP-FL) with γ=0.1. (**a**) MNIST; (**b**) Fashion-MNIST; (**c**) CIFAR-10.

**Model Accuracy:** First, Figure 5 shows that the accuracy of SOLO is significantly lower than FedAvg, validating the effectiveness of FL. Compared to other methods, TopkSDP-FL shows superior performance across all datasets at γ=0.1. In the MNIST dataset, TopkSDP-FL consistently outperforms other approaches, achieving a test accuracy of 96.61%, which is a 1.67% improvement over FedAvg’s 94.94%, 1.77% over FedProx’s 94.84%, and 2.39% higher than FedADMM’s 94.22%. The accuracy of other methods, such as SCAFFOLD and MOON, does not exceed 95%. In the Fashion-MNIST dataset, TopkSDP-FL achieves an accuracy of 84.83%, surpassing FedAvg by 2.02%, FedProx by 1.9%, and FedADMM by 2.01%. It also outperforms SCAFFOLD and MOON, which recorded accuracies of 82.98% and 81.88%, respectively. In the CIFAR-10 dataset, TopkSDP-FL attains an accuracy of 62.59%, 3.61% higher than FedAvg, 2.24% higher than FedProx, and 2.54% higher than FedADMM. During the early stages of training, TopkSDP-FL exhibits faster convergence compared to FedAvg, FedProx, and FedADMM, maintaining a higher level of accuracy after the number of communication rounds reaches a certain threshold. This shows that TopkSDP-FL effectively addresses data heterogeneity while excelling in model convergence speed and global accuracy.

This is mainly owing to our proposed local loss function based on contrastive learning, which effectively strengthens the coordination between the local model and the global model and alleviates the problem of data heterogeneity, thus accelerating the convergence and improving the global performance of the model.

**Figure 6:**
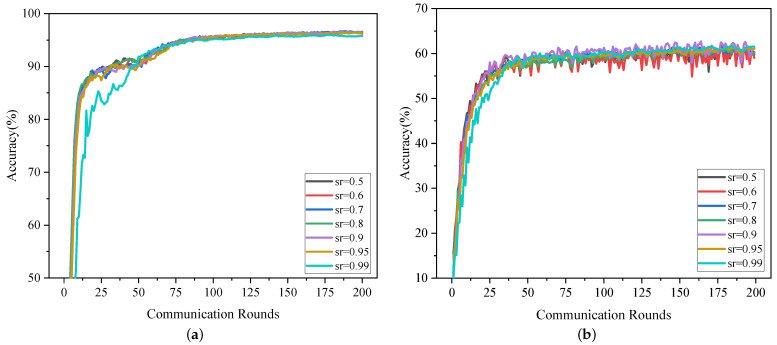
The test accuracy of different sparsity sr with γ=0.1. (**a**) MNIST; (**b**) CIFAR-10.

**Communication Efficiency:** TopkSDP-FL also significantly reduces communication overhead while maintaining high model accuracy, as shown in Figure 6. Experiments conducted on the MNIST and CIFAR-10 datasets evaluate the impact of the global sparsity rate (sr) on communication efficiency. With a local sparsity rate (sl) of 0.9, TopkSDP-FL achieves a reduction of 94. 5% in communication overhead at sr=0.99. Specifically, for MNIST, TopkSDP-FL achieves an accuracy of 96.6% at sr=0.99, compared to 94.4% for uncompressed FedAvg. On CIFAR-10, TopkSDP-FL achieves an accuracy of 61.4%, a 2% improvement over FedAvg’s 58.98%.

**Figure 7:**
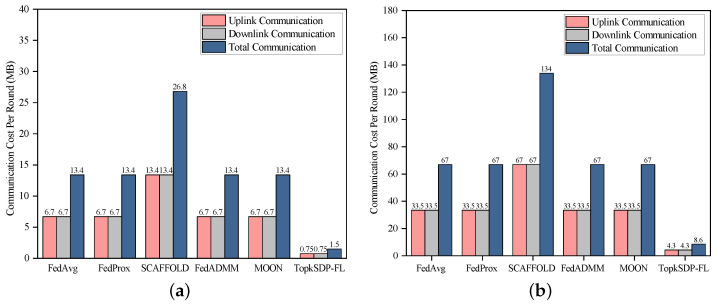
Communication cost per round for different algorithms (i.e., FedAvg [2], FedProx [6], SCAFFOLD [7], FedADMM [25], MOON [8], and TopkSDP-FL). (**a**) MNIST; (**b**) CIFAR-10.

Table 1 shows the communication overhead at different sr. When sr=0.99, the communication cost for the MNIST dataset is reduced to 0.96 MB, which is about 93% less compared to 13.4 MB without sparsification. For the CIFAR-10 dataset, the communication cost is 5.7 MB when sr=0.99, compared to 67.02 MB for the FedAvg, which is a 92% reduction. Moreover, Figure 7 shows the difference in communication overhead between the different algorithms on the MNIST and CIFAR-10 datasets. The communication cost for FedAvg, FedProx, FedADMM, and MOON on the MNIST dataset is 13.4 MB (6.7 MB uplink and 6.7 MB downlink). For SCAFFOLD, it goes up to 26.8 MB, mainly due to the introduction of control variables. TopkSDP-FL has the lowest communication overhead of 1.5 MB. On the CIFAR-10 dataset, the communication overhead of FedAvg, FedProx, and MOON increases to 134 MB (67 MB upstream, 67 MB downstream). The communication overhead of FedAvg, FedProx, and MOON rises to 134 MB (67 MB uplink, 67 MB downlink), while TopkSDP-FL remains the lowest at 8.6 MB, demonstrating its optimization of communication efficiency.

From the above experimental results, we can see that TopkSDP-FL significantly reduces communication overhead while maintaining high model accuracy. The key to this effect depends on our proposed bidirectional top-*k* sparsification technique. This technique optimizes communication efficiency by reducing the communication overhead in both the uplink and downlink. Moreover, since the sparsity of the model does not significantly affect its performance, we can flexibly control the balance between bandwidth consumption and model performance by adjusting the sparsity rate (sr). In this way, TopkSDP-FL is particularly suitable for bandwidth-constrained IoT and edge computing scenarios, ensuring efficient model updates and accurate inference performance despite limited bandwidth.

**Figure 8:**
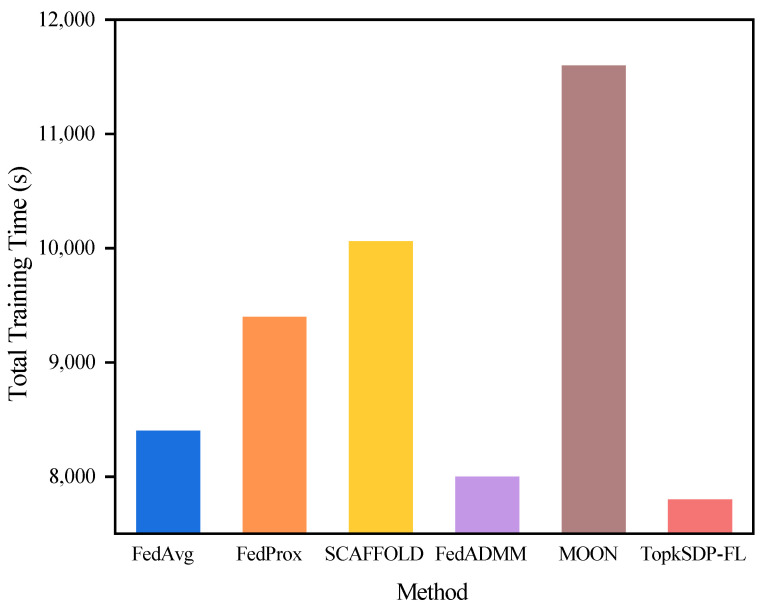
Comparison of total training time on the MNIST dataset.

**Computational Overhead:** We experimentally recorded the total training time of different algorithms on the MNIST dataset and graphically show the total training time of each algorithm, as shown in Figure 8. According to the experimental results, the TopkSDP-FL method requires the shortest total training time among all methods. This result is mainly due to the sparse randomization mechanism we introduced. This mechanism works by sparsifying the model parameters, keeping only the most important K non-zero parameters, and setting the rest to zero. During the model update process, only the non-zero parameters are passed and computed, which greatly reduces the number of parameters that need to be processed, and thus effectively reduces the communication overhead. In this way, many zero parameters can be skipped during the computation process, which avoids invalid computation and significantly improves computational efficiency. As a result, TopkSDP-FL is able to reduce the total training time while ensuring efficient training significantly.

### 5.3. Robustness to Data Heterogeneity

In this subsection, we evaluate the performance of various FL algorithms by adjusting the parameter γ to simulate different levels of data heterogeneity. The experimental results demonstrate that TopkSDP-FL achieves excellent performance with consistently high accuracy across the three datasets (MNIST, Fashion-MNIST, and CIFAR-10).

As illustrated in Table 2, in the low-heterogeneity scenario (γ=0.5), the performance differences among the algorithms are relatively small. However, TopkSDP-FL still maintains a slight advantage, achieving 97.65%, 88.74%, and 64.23% accuracy on the MNIST, Fashion-MNIST, and CIFAR-10 datasets, respectively. Compared to FedAvg, it improves accuracy by 0.19%, 0.13%, and 3.6%, while compared to FedProx, the improvements are 0.18%, 0.18%, and 3.1%, respectively. FedADMM also performs well, but its accuracy remains slightly below that of TopkSDP-FL, particularly on CIFAR-10. At this point, due to low data heterogeneity, all methods exhibit stable training, leading to close model convergence.

However, when the heterogeneity increases to γ=0.1, the performance gap begins to widen gradually. TopkSDP-FL achieves 96.61% accuracy on MNIST, which is 1.67%, 1.77%, and 2.39% higher than FedAvg, FedProx, and FedADMM, respectively. On Fashion-MNIST, TopkSDP-FL attains 84.83%, outperforming FedAvg, FedProx, and FedADMM by 2.02%, 1.9%, and 2.01%, respectively. A similar trend is observed on CIFAR-10, where TopkSDP-FL reaches 62.59%, improving upon FedAvg and FedProx by 3.61% and 2.24%, and FedADMM by 2.54%. Additionally, TopkSDP-FL still outperforms SCAFFOLD and MOON, particularly on Fashion-MNIST, where SCAFFOLD and MOON achieve 82.98% and 81.88%, respectively. When the data heterogeneity further intensifies (γ=0.01), TopkSDP-FL remains robust, achieving 92.46%, 82.06%, and 53.15% accuracy on MNIST, Fashion-MNIST, and CIFAR-10, respectively. Compared to FedAvg, these results represent improvements of 15.07%, 6.68%, and 5.62%, while compared to FedProx, the accuracy gains are 12.34%, 6.84%, and 2.22%, respectively. FedADMM, although exhibiting some improvements over FedAvg and FedProx, does not surpass TopkSDP-FL, particularly on CIFAR-10, where its accuracy remains 4.89% lower. Notably, methods like SCAFFOLD and MOON show no substantial performance gains over FedAvg, particularly in high-heterogeneity conditions.

In conclusion, TopkSDP-FL demonstrates strong performance across different levels of data heterogeneity. While all methods perform similarly in low-heterogeneity settings, the advantages of TopkSDP-FL become more apparent as heterogeneity increases. Compared to FedAvg, FedProx, FedADMM, SCAFFOLD, and MOON, our method consistently achieves higher accuracy, especially in extreme heterogeneity conditions, showcasing superior robustness, adaptability, and model convergence.

### 5.4. Privacy Budget and Sparsity Analysis

**Figure 9:**
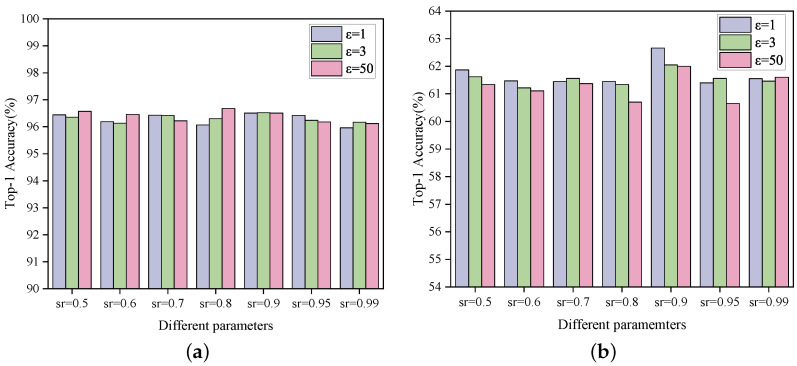
The test accuracy under different sparsity levels (sr) and privacy budgets (ϵ) with γ=0.1. (**a**) MNIST; (**b**) CIFAR-10.

This subsection investigates how the privacy budget ϵ and the global sparsity rate sr influence model performance. The privacy budget ϵ achieves a trade-off between privacy protection and model utility: smaller values of ϵ enhance privacy protection but may lead to a degradation of model performance, while larger values of ϵ enhance model utility but weaken privacy protection. We set ϵ to 1, 3, and 50, and show the model accuracy performance of TopkSDP-FL under different combinations of ϵ and sr in Figure 9.

As can be seen from Figure 9, unlike what is expected from traditional DP schemes, a larger privacy budget ϵ does not always result in higher model accuracy for the same sparsity sr. This is mainly because our proposed sparseRand method achieves DP preservation by randomizing only the important parameters. This results in less noise being added to each parameter, which in turn has less impact on the model performance. For example, when the sparsity rate sr=0.5, the model accuracy for ϵ=50 is lower than the corresponding value for ϵ=1 for both the MNIST dataset and the CIFAR-10 dataset. This shows that TopkSDP-FL can effectively reduce the accuracy loss caused by noise accumulation during the sparsification process, thus alleviating the problem of model performance degradation due to noise increase in the traditional DP scheme.

Moreover, unlike baseline methods such as FedAvg, FedProx, and SCAFFOLD, which lack any privacy-preserving mechanisms, TopkSDP-FL achieves a balance between privacy protection and model utility. This highlights the advantage of our method in handling data privacy concerns while maintaining superior model performance.

### 5.5. The Effects of Hyperparameters

In this subsection, we focus on the impact of two hyperparameters on model performance in our proposed loss function. These two hyperparameters are α and β, which control how much the model prefers the global model and the local model of the previous round during training. Specifically, the larger α is, the closer the model is to the global model, while the larger β is, the greater the distance between the local model and the previous round of modeling, reducing the dependence on the state of the historical model.

To better understand the impact of these hyperparameters on model performance, we first fix α=0.001 and evaluate the effect of different values of β (0.001, 0.005, and 0.01) on model accuracy. As shown in Table 3, across all three datasets, the accuracy of TopkSDP-FL remains relatively stable under different β values and consistently outperforms FedAvg. For instance, on the MNIST dataset, the accuracy reaches 96.61% at β=0.005, which is 1.67% higher than that of FedAvg. Similarly, the accuracy on the Fashion-MNIST and CIFAR-10 datasets also shows a significant improvement, demonstrating the superior performance of TopkSDP-FL across different datasets.

Therefore, in exploring the effect of α on model performance, we keep β=0.005 and show the change in accuracy for different α values (0.001, 0.005, and 0.01). As shown in Table 4, according to the experimental results, as the value of α increases, the model accuracy does not improve significantly, remains stable, and consistently outperforms FedAvg. In particular, the model performs best when α=0.001, reaching 96.61% for MNIST, 84.83% for Fashion-MNIST, and 63.04% for CIFAR-10. These results show that the combination of α=0.001 and β=0.005 exhibits good accuracy on all three datasets, further demonstrating the stability and generalizability of this hyperparameter combination across different scenarios.

## 6. Conclusions

This study proposes TopkSDP-FL, an FL framework addressing key challenges in non-IID and resource-constrained settings. To alleviate the effects of data heterogeneity, we propose a new regularization technique that aligns local updates more effectively with the global objective, thereby enhancing model performance. We achieve communication efficiency through a bidirectional top-*k* sparsification strategy, which considerably reduces both uplink and downlink communication overheads. Additionally, we implement double privacy amplification by integrating shuffling and subsampling techniques. By incorporating layer-wise parameter shuffling, this approach effectively reduces the risk of privacy budget explosion and enhances the anonymity of local models. Experimental results confirm the strong performance of TopkSDP-FL, demonstrating its ability to achieve a balanced trade-off among accuracy, communication efficiency, and privacy preservation.

Our TopkSDP-FL method has broad application potential in IoT devices, smart healthcare, and smart city infrastructure, enhancing model performance while ensuring privacy protection. In smart healthcare, it facilitates collaborative training among hospitals, preserving patient privacy while effectively handling heterogeneous medical data. By incorporating a bidirectional communication mechanism and hierarchical parameter shuffling, our approach reduces communication overhead and mitigates model drift caused by data heterogeneity through regularization. TopkSDP-FL offers an efficient and privacy-preserving collaborative solution. Since our algorithms were not designed with sufficient consideration of fairness between users and personalized differential privacy, in our future work, we will explore more equitable training mechanisms and more advanced privacy-preserving methods to enhance the adaptability and scalability of the framework. In addition, we will investigate how to combine TopkSDP-FL with machine learning paradigms such as migration learning and reinforcement learning to address more complex application scenarios.

## Figures and Tables

**Table 1:** Communication costs with different sparsity sr.

Dataset	sr = 0.5	sr = 0.6	sr = 0.7	sr = 0.8	sr = 0.9	sr = 0.95	sr = 0.99
MNIST	4.2	3.5	2.8	2.2	1.5	1.2	0.96
CIFAR-10	21.6	18.3	15.1	11.8	8.6	6.9	5.7

Notes: The total communication overhead per round without sparsification is 13.4 MB for MNIST and 67.02 MB for CIFAR-10.

**Table 2:** The test accuracy of six methods (i.e., FedAvg [2], FedProx [6], SCAFFOLD [7], MOON [8], FedADMM [25], TopkSDP-FL and SOLO) with γ from {0.5, 0.1, 0.01}.

Dataset	γ	FedAvg	FedProx	SCAFFOLD	MOON	FedADMM	SOLO	TopkSDP-FL
MNIST	0.5	97.46	97.47	97.48	97.49	97.60	72.46	**97.65**
	0.1	94.94	94.84	94.58	94.32	94.22	40.57	**96.61**
	0.01	77.39	80.12	81.27	61.61	74.08	10.88	**92.46**
Fashion-MNIST	0.5	88.61	88.56	88.48	88.59	88.32	68.72	**88.74**
	0.1	82.81	82.93	82.98	81.88	82.84	40.39	**84.83**
	0.01	75.38	75.22	75.72	75.24	74.07	16.49	**82.06**
CIFAR-10	0.5	60.63	61.13	61.50	61.28	62.18	35.47	**64.23**
	0.1	58.98	60.35	59.61	59.38	60.05	26.97	**62.59**
	0.01	47.53	50.93	51.04	42.78	49.26	13.06	**53.15**

**Table 3:** The test accuracy of TopkSDP-FL with β from {0.001, 0.005, 0.01}, where α=0.001.

β	MNIST	Fashion-MNIST	CIFAR-10
β=0.001	95.89	83.92	62.59
β=0.005	**96.61**	**84.83**	**63.04**
β=0.01	96.12	84.11	61.89

**Table 4:** The test accuracy of TopkSDP-FL with α from {0.001, 0.005, 0.01}, where β=0.005.

α	MNIST	Fashion-MNIST	CIFAR-10
α=0.001	**96.61**	**84.83**	**63.04**
α=0.005	96.19	84.71	62.34
α=0.01	96.26	84.76	62.05

## Data Availability

All experiments from this paper were performed on public datasets.

## Abbreviations

The following abbreviations are used in this manuscript:

FL	Federated learning
non-IID	Non-Independent and Identically Distributed
DP	Differential privacy
SDP	Shuffled differential privacy
IoT	Internet of Things
